# Lessons Learned From Focus Groups and Community Education Provision in the African American Community

**DOI:** 10.1093/geroni/igab046.1786

**Published:** 2021-12-17

**Authors:** Robbin Frazier

**Affiliations:** University of Minnesota, University of Minnesota/Minneapolis, Minnesota, United States

## Abstract

The present symposium will synthesize themes derived from eight different projects designed to better understand dementia in Minnesota (MN) within the African American community (where individuals are disproportionately susceptible to dementia and the tolls of dementia care). These projects included focus groups, community outreach, community education, networking with aging service providers, and community forums. Projects were funded by and conducted in partnership with the MN Department of Human Services, the Alzheimer’s Association, the MN Board on Aging, and the MN Leadership Council on Aging’s Diverse Elders Coalition. Themes included the unique ways that African American elders share their viewpoints and the importance of faith-based outreach. Another major theme, which connects to the other symposium talks, was the importance of three S’s: Stigma, Shame, and Silence as cultural considerations in the African American, West African, and Latino/a/x/e communities as they apply to access to information and training to better understand AD/ADRD.

